# Effectiveness of weight-loss prevention with continual nutrition counseling in postoperative outpatients with stage IA and IB gastric cancer

**DOI:** 10.1371/journal.pone.0292920

**Published:** 2023-10-19

**Authors:** Asami Matsushita, Eiji Nakatani, Chika Shibasaki, Saaya Tanabe, Nanami Iwasaki, Tomoko Okamura, Aya Nozaki, Saeko Aoshima, Reiko Takahashi, Masaya Watannabe, Toshio Shimada

**Affiliations:** 1 Department of Nutrition, Shizuoka General Hospital, Shizuoka, Japan; 2 Graduate School of Public Health, Shizuoka Graduate University of Public Health, Shizuoka, Japan; 3 Research Support Center, Shizuoka General Hospital, Shizuoka, Japan; 4 Department of Gastroenterological Surgery, Shizuoka General Hospital, Shizuoka, Japan; Stanford University School of Medicine, UNITED STATES

## Abstract

Outpatient nutritional counseling by a registered dietitian is often performed to prevent weight loss, but evidence supporting this practice is insufficient. In this study, we aimed to clarify the effectiveness of four-time outpatient nutritional counseling in weight-loss prevention compared with conventional intervention limited to one-time nutritional counseling. This study was designed as a retrospective cohort study. The target population was postoperative patients with stage IA and IB gastric cancer. Groups that received one-time and four-time nutritional counseling included patients who underwent gastrectomy from May 2014 to April 2017 and May 2017 to December 2019, respectively. The one-time group received counseling at discharge; the four-time group received counseling at discharge, at the first outpatient visit, and at 3 and 6 months postoperatively. There were 58 patients in the one-time group and 27 patients in the four-time group, with a significant difference in length of hospital stay (p = 0.042). Thirty-six patients (62.1%) in the one-time nutritional counseling group and 12 (44.4%) in the four-time group had a weight loss of 5% or more from hospital discharge to 6 months postoperatively. The adjusted risk ratio for the effectiveness of four counseling sessions compared with one session was 0.69 (95% confidence interval 0.35–1.34). In subgroup analysis, the effect of nutritional guidance was greater for patients with body mass index ≥23 kg/m2, but this depended on the outcome and number of cases, and there was no essential difference between the groups. In postoperative patients with stage IA and stage IB gastric cancer, four sessions of outpatient nutrition counseling may be not superior to one counseling session in preventing weight loss.

## Introduction

Gastric cancer is the fifth most common human malignancy and the third most frequent cause of cancer-related death worldwide. Incidence rates are markedly elevated in parts of East Asia, such as in Mongolia, Japan, and the Republic of Korea, which has the highest rates worldwide in both sexes [1]. Weight loss after gastrectomy in patients with gastric cancer is accompanied by decreased oral intake, resulting in gastric obstruction; decreased or absent gastric juice secretion; impaired digestion; poor absorption of nutrients; and exocrine pancreatic insufficiency. Some researchers have reported that >10% weight loss within the previous 6 months is observed in 15% of patients diagnosed with gastroesophageal cancer. Malnutrition occurs in up to 80% of patients with advanced-stage gastric cancer [2, 3]. Therefore, weight loss is an essential index related to quality of life (QOL) in postoperative outpatients with gastric cancer [3–6]. Inappropriate weight loss that is not fat loss, with loss of muscle mass, in patients with cancer is associated with poor prognosis, unlike some diseases in which weight loss may be desirable [7–10].

Currently, outpatient nutritional counseling by a registered dietitian for patients with cancer is usually performed to prevent weight loss; however, the evidence is insufficient regarding this practice [11–15]. Previously, postoperative nutritional counseling at our hospital was only provided once at discharge as group nutritional counseling, and outpatient nutritional counseling was only provided for patients in whom it was deemed necessary by their physician. However, since May 2017, nutrition counseling by a dietitian is provided four times: at the time of discharge, at the first outpatient visit, 3 months after surgery, and 6 months after surgery.

Against this background, in the present study, we aimed to clarify the effect of continual (four-session) outpatient nutritional counseling for weight loss prevention in patients with gastric cancer, in comparison with conventional intervention limited to one-time nutritional counseling.

## Materials and methods

### Study design and comparison groups

This was a retrospective cohort study, and data were collected in a review of the medical records. Groups that received one-time or four-time outpatient nutrition counseling included patients who received counseling at discharge from gastrectomy between May 2014 and April 2017 and between May 2017 and December 2019, respectively. The one-time counseling group received nutritional counseling at discharge; the four-time counseling group received nutritional counseling at discharge, at the first outpatient visit and at 3 and 6 months postoperatively.

To eliminate the effect of adjuvant chemotherapy, we excluded patients with stage II or higher disease and those with a history of other cancer. Patients who did not receive at least three counseling sessions in the four-time counseling group were also excluded.

### Nutrition counseling to prevent postoperative weight loss

In the four-session group, nutritional counseling was provided under a physician’s direction at discharge from the hospital, at the first outpatient visit, and at 3 and 6 months after surgery. An original questionnaire was used, designed to capture individual nutritional characteristics through essential items for tailored nutritional guidance. This tool was honed through repeated use in individual consultations, leading to refinements and modifications to its current format. Items on the questionnaire included items on the questionnaire included postoperative discomfort (such as early dumping syndrome or late dumping syndrome), chewing and swallowing functions, eating habits (number of meals, whether meals are eaten, number of times food is chewed, the content of meals), living environment, the burden on cooking staff, and performance status. Based on the questionnaire responses, semi-structured and customized counseling sessions were conducted. Nutritional counseling was tailored based on these responses.

A dietitian advised each patient to chew slowly and well, to eat smaller portions, and to eat more frequently to prevent dumping syndrome and compensate for decreased digestion and stomach acid secretion capacity. Additionally, to prevent weight loss, we checked patients’ usual food intake, advised them regarding correct eating habits, and encouraged them to take oral nutritional supplements.

For the one-session group, nutritional counseling was conducted in a similar manner but was provided solely at the time of discharge from the hospital, using the guidance and methods from the four-session group.

### Weight loss rate from hospital discharge to 6 months

To explore the effectiveness of nutritional counseling in preventing weight loss, the presence or absence of a weight loss rate of ≥5% from hospital discharge to 6 months postoperatively was used as the primary outcome. Patients’ weight was measured at the first outpatient visit, at discharge, and at 6 months. The weight change (%) was calculated by dividing the weight at 6 months postoperatively minus the weight at discharge by the weight at discharge. There is consensus among the dietitians and surgeons who participated in this study that weight loss greater than 5% is clinically significant; therefore, this primary outcome was considered appropriate.

### Variables at discharge

Baseline variables at the time of discharge were age, sex, body mass index (BMI), weight, percentage weight loss from the preoperative period to discharge, the extent of gastrectomy, surgical approach, stage of the disease, pretreatment, diabetes, chronic kidney disease, length of hospital stay, and blood biochemical parameters (albumin, C-reactive protein, total protein, hemoglobin, total lymphocytes). The gastrectomy extent was classified as total gastrectomy (TG), proximal gastrectomy (PG), distal gastrectomy (DG), or pylorus-preserving gastrectomy (PPG). Pretreatment mainly comprised endoscopic mucosal dissection.

### Statistical analysis

Categorical and continuous variables are summarized as frequency (percentage) and mean ± standard deviation, respectively. Fisher’s exact test was used to compare groups for categorical variables and the *t*-test for continuous variables. Univariable and multivariable Poisson regression analyses were also performed to calculate risk ratios, 95% confidence intervals (CIs), and p-values. The variables with p<0.1 in the background table (Table 1) and variables with p<0.1 in univariable analysis (Table 2), which were confounder candidates, were entered into the multivariable regression model. As a sensitivity analysis, we also performed univariable and multivariable logistic regression analyses, which were performed to calculate odds ratios, 95% confidence intervals (CIs), and p-values. To identify subgroups for which nutritional counseling was effective, interaction tests between nutritional counseling and stratified variables were performed in the multivariable Poisson regression model with the subgroup-indicated variable, treatment group, their interaction term, and other confounder candidates as covariates. We used IBM SPSS version 22 (IBM Corp., Armonk, NY, USA) and EZR ver. 1.36 for all statistical analyses. A p-value <0.05 was considered statistically significant.

**Table 1:** Patient characteristics.

Variable	Category (Unit)	One-time nutritional counseling group	Four-times Nutritional counseling group	P-value
		(n = 58)	(n = 27)	
Age	(Year)	68.9±10.1	64.1±12.2	0.060
Sex	Male	35	16	
	Female	23	11	>0.999
Weight at discharge	(kg)	54.9±9.4	58.6±12.0	0.124
BMI at discharge	(kg/m^2^)	21.4±3.1	22.4±2.8	0.152
Percent reduction from preoperative to discharge	(%)	-4.9±2.9	-4.7±3.2	0.827
Gastrectomy extent	TG and PG	16 (28)	7 (26)	>0.999
	DG and PPG	42 (72)	20 (74)	
Surgical approach	Laparotomy	6 (10)	4 (15)	0.719
	Laparoscopic/robotic	52 (90)	23 (85)	
Stage	I A	45 (78)	21 (78)	>0.999
	I B	13 (22)	6 (22)	
Pretreatment	No	51 (88)	20 (83)	0.723
	Yes	7 (12)	4 (17)	
Diabetes mellitus	No	50 (86)	23 (85)	>0.999
	Yes	8 (14)	4 (15)	
Chronic kidney disease	No	57 (98)	27 (100)	>0.999
	Yes	1 (2)	0	
**Duration of hospitalization**	**(days)**	**12.4±3.8**	**14.6±6.0**	**0.042**
**Albumin at discharge**	**(g/dl)**	**3.2±0.4**	**3.4±0.4**	**0.092**
C-reactive protein at discharge	(g/dl)	3.7±3.0	2.6±2.5	0.105
Total protein at discharge	(g/dl)	7.0±0.6	7.0±0.4	0.794
Hemoglobin at discharge	(g/dl)	12.1±1.5	12.4±1.5	0.439
Total lymphocytes at discharge	(/μl)	1206.3±380.9	1211.6±306.3	0.951

BMI, body mass index; TG, total gastrectomy; PG, proximal gastrectomy; DG, distal gastrectomy; PPG, pylorus-preserving gastrectomy.

**Table 2:** Univariable Poisson regression analysis for weight loss from hospital discharge to 6 months postoperatively.

Variable	Category (Unit)	Weight loss of <5% Group	Weight loss of ≥5% Group	Univariable logistic model		
		(n = 37)	(n = 48)	RR	95% CI	p-value
Nutritional counseling group	One-time	22	36	1	–	–
	Four-times	15	12	0.73	0.38–1.42	0.361
Age	(Age)	64.9±11.1	69.3±10.6	1.01	0.99–1.04	0.292
Sex	Male	24	27	1	–	–
	Female	13	21	0.93	0.52–1.68	0.812
Weight at discharge	(kg)	55.3±11.7	56.7±9.4	1.01	0.98–1.04	0.535
BMI at discharge	(kg/m2)	20.9±3.5	22.3±2.5	1.04	0.94–1.14	0.470
Weight loss rate from surgery to hospital discharge	(%)	-5.7±3.0	-4.2±2.8	0.94	0.85–1.04	0.206
Gastrectomy extent	TG and PG	6	17	1	–	–
	DG and PPG	31	31	0.60	0.33–1.08	**0.086**
Surgical approach	Laparotomy	6	4	1	–	–
	Laparoscopic/robotic	31	44	1.43	0.51–3.99	0.491
Stage	I A	29	37	1	–	–
	I B	8	11	1.19	0.62–2.29	0.6013
Pretreatment	None	29	42	1	–	–
	Yes	7	4	0.81	0.32–2.04	0.651
Diabetes	No	30	43	1	–	–
	Yes	7	5	0.89	0.38–2.10	0.790
Chronic kidney disease	No	36	48	1	–	–
	Yes	1	0	1.83	0.25–13.24	0.551
Duration of hospitalization	(days)	14.2±5.7	12.3±3.6	1.00	0.94–1.06	0.988
Albumin at discharge	(g/dl)	3.4±0.4	3.3±0.4	0.72	0.29–1.79	0.479
C-reactive protein at discharge	(mg/dL)	2.6±2.6	3.9±3.0	1.04	0.95–1.14	0.409
Total protein at discharge	(g/dl)	7.0±0.4	7.0±0.6	0.99	0.57–1.71	0.973
Hemoglobin at discharge	(g/dl)	12.2±1.5	12.2±1.5	0.96	0.79–1.16	0.649
Total lymphocytes at discharge	(/μl)	1203.2±393.6	1211.7±330.4	1.00	1.00–1.00	0.999

CI, confidence interval; RR, risk ratio; NE, not evaluable; BMI, body mass index; TG, total gastrectomy; DG, distal gastrectomy; PG, proximal gastrectomy; PPG, pylorus-preserving gastrectomy.

### Ethics

This study complied with the Ethical Principles for Medical Research Involving Human Subjects published by the Ministry of Health, Labour and Welfare and the Ministry of Education, Culture, Sports, Science, and Technology. To obtain data from the medical records, we disclosed the purpose and methods of this study to the study patients on the home-page of the Shizuoka General Hospital website and guaranteed their right to refuse participation in the study. Identifiable information, such as individual names, etc., was deleted from the obtained data and only the anonymized data were used for this study. The Ethics Committee of Shizuoka Prefectural General Hospital approved the study (no. SGHIRB#2018074, 2018).

## Results

In this study, 58 patients were included in the one-time nutritional counseling group and 27 patients were included in the four-time nutritional counseling group. The patient background at discharge is shown in Table 1; there was a significant difference in the length of hospital stay (p = 0.042).

The median (min–max) time from discharge to the first outpatient visit and to the 6-month time point was 14 (5–38) days and 176 (121–204) days, respectively. The number of patients with weight loss of 5% or more from hospital discharge to 6 months postoperatively was 36 (62.1%) in the one-time nutritional counseling group and 12 (44.4%) patients in the four-time group. Univariable Poisson regression analysis showed that pretreatment, but not nutritional counseling groups, predicted ≥5% weight loss in association with BMI at discharge and percent weight loss from the preoperative period to discharge (Table 2).

The crude risk ratio (95% CI) for the effectiveness of four-time counseling compared with one-time counseling was 0.73 (0.38–1.42) (Table 2). In multivariable Poisson regression analysis, the adjusted risk ratio (95% CI) was 0.77 (0.35–1.68) (Table 3). In a sensitivity analysis, the crude odds ratio (95% CI) for the effectiveness of four-time counseling compared with one-time counseling was 0.49 (0.19–1.23) (S1 Table), and the adjusted odds ratio (95% CI) was 0.62 (0.16–2.40) (S2 Table).

**Table 3:** Multivariable Poisson regression analysis for weight loss from hospital discharge to 6 months postoperatively.

Variable	Category (Unit)	Multivariable logistic analysis		
		aRR	95% CI	p-value
Nutritional counseling group	One-time	1		
	Four-times	0.77	0.35–1.68	0.511
Duration of hospitalization	(days)	0.98	0.91–1.06	0.681
Albumin at discharge	(%)	0.77	0.29–2.03	0.602
Gastrectomy extent	TG and PG	1	–	–
	DG and PPG	0.62	0.28–1.36	0.237

CI, confidence interval; aRR, adjusted risk ratio; TG, total gastrectomy; DG, distal gastrectomy; PG, proximal gastrectomy; PPG, pylorus-preserving gastrectomy.

Among subgroup analyses in S3 Table, the adjusted risk ratios for nutritional counseling with BMI <23 kg/m^2^ and BMI ≥23 kg/m^2^ were respectively 3.32 (0.23–47.02) and 0.09 (0.01–1.26), and the result of their corresponding interaction test was p = 0.014. No other subgroup analyses had significant interaction tests.

## Discussion

In this study, we examined the effectiveness of continual outpatient nutrition counseling in preventing weight loss among 85 postoperative patients with stage IA and IB gastric cancer. The results showed that overall, intensive outpatient nutrition counseling intervention was not effective in preventing weight loss. The preventive effect of outpatient nutrition counseling on weight loss from discharge to 6 months was greater in patients with higher BMI.

Hye et al. [14] investigated the effects of intensive education (IE) during the first 3 months after hospital discharge on nutritional status, diet quality, and QOL compared with conventional education (CE) in Korean 65 patients who underwent gastrectomy. The IE group received seven sessions of nutrition education; these patients reported improvements in self-efficacy and food satisfaction 3 weeks after discharge, in comparison with the CE group that received a single session of nutrition education. However, the two groups had no differences in weight loss [14]. Although weight loss was used as an outcome in that study, nutritional counseling might have contributed to self-efficacy and food satisfaction [14]. Thus, increasing the frequency of nutrition education as a preventive measure against weight loss cannot be expected to have the same effect as post-discharge nutrition instruction.

More than simple nutritional counseling may be needed for weight maintenance in postoperative patients with cancer. In a randomized controlled trial in a Korean cancer center, 56 patients with stage I to III gastric cancer who underwent gastrectomy were separated into two groups, either following a patient participation-based diet and usual care or receiving two in-person and two telephone interventions. At 12 weeks, there was no significant difference in weight change [15]. In a randomized controlled trial of 358 patients receiving palliative chemotherapy for gastrointestinal and non-small cell lung cancer or mesothelioma, there was no difference in weight after 1 year of follow-up between groups that received no intervention and dietary advice [11]. Like these studies, the results of the present study did not show any effect in deterring weight loss 6 months after hospital discharge. However, two studies with optimization of individual energy and protein needs [13] and individual nutritional measurements [12], together with nutritional counseling, reported that participants experienced weight maintenance and weight gain. Therefore, providing individual nutritional measurement, prediction of nutritional requirements, and nutritional counseling may be needed in these patients.

The results of this study of 85 patients showed that the point estimate of the adjusted odds ratio was 0.69, indicating that the four nutrition counseling sessions group may have been superior to the one nutrition counseling session group. However, the corresponding confidence interval included 1, which was not statistically significant. Here, in a logistic regression in which the response and exposure variables were binary without considering confounding, the sample size was 85 cases as in this study, the <5% weight loss rate for the one nutrition counseling group was 0.379, the proportion of the four-nutrition counseling group was 0.318, the odds ratio for the four times group compared to the one times group was 0.69 and the significance using a two-tailed Wald test with a significance level of 5%, the power is 8.5%. In other words, the type 2 error rate for the test was 91.5% and could not be detected. However, the determination of no effect by the test in this study supports the results of three previous randomized controlled trials [11, 14, 15] related to the effect of education on patients. We report these results for this reason, without increasing the number of cases for the purpose of detecting statistical significance.

It has been reported that ghrelin administration suppresses weight loss in TG [16]. With TG and PG, the appetite hormone ghrelin is not secreted, so nutritional intake does not increase, and nutritional counseling may not be sufficient. In the future, weight loss might be prevented by prescribing Chinese herbal medicines with ghrelin-like effects, such as Rikunshi-to [17], to stimulate ghrelin secretion from sources other than the upper part of the stomach. However, avoiding TG and PG should be considered, when possible, as a measure to prevent weight loss [18, 19].

In our study, the weight loss rate after discharge, when outpatient nutrition counseling was initiated, was higher in patients with relatively low weight loss from the preoperative period to discharge. Therefore, it is desirable to have a method of intervening in this population such that nutritional guidance will positively affect weight loss. It is also important to investigate why these individuals did not lose weight during hospitalization.

There are several limitations to this study. First, although the research period was extensive, this was a single-center, small-sample, retrospective cohort study; future validation in a large, multicenter, randomized controlled trial is necessary. Second, while we estimated the average causal effect, our adjustments might have accounted for only some confounders, potentially overlooking unobserved ones. Specifically, unadjusted age (p = 0.06 in Table 1) and duration of hospitalization (p = 0.042 in Table 1) were either significant or nearly significant, implying potential imbalances in patient demographics for effect comparison. However, to prevent Poisson regression model collapse, we refrained from adjustments via multivariate regression models with numerous covariates. Third, the one- and four-session counseling interventions were conducted in distinct time frames. Because the duration of counseling in each group was relatively short and there was no interval between the end of the one-session intervention and beginning of the four-session intervention, the medical environment and content of nutritional counseling were likely similar. However, we acknowledge that the variations in counseling durations and intervention specifics could have influenced the results, introducing a potential source of bias.

Fourth, we collected nutritional data through a questionnaire and direct patient queries. We did not record detailed data on patients’ energy, nutrient intake, recommended supplements, or the types of supplements taken, so we could not determine whether their weight loss was due to inadequate food intake and supplementation or digestive problems. In the future, it will be necessary to employ data quantification methods, such as the Omaha System [20], to quantitatively describe the standard nutritional guidance and the actual nutritional guidance provided within the study. Furthermore, the differences in the dietary interventions compared may introduce potential bias in the effect comparisons between the two groups. Fifth, the limitations of subgroup analysis include low statistical power due to small sample sizes and the need for cautious interpretation owing to multiple tests conducted. Nevertheless, these analyses can offer valuable insights for identifying populations that may benefit from targeted nutritional guidance.

Sixth, our study, while offering valuable insights, encompasses patients who underwent both total gastrectomy and distal gastrectomy. The two groups are expected to experience differing levels of nutritional challenges and weight loss, with the total gastrectomy group often requiring more substantial nutritional support. This mixed patient cohort could introduce some variability in our results. Future studies are recommended to conduct separate analyses for these two surgical groups, which could provide a more precise understanding of the effects of nutritional interventions specific to each surgery type. However, there was no significant difference in the type of surgical procedure between the intervention and control groups (Table 1). Whereas this strengthens the internal validity of our study, there is still a potential for heterogeneity.

## Conclusion

In postoperative patients with stage IA and stage IB gastric cancer, the effects of four outpatient nutrition counseling sessions may be not superior to those of one nutrition counseling session in preventing weight loss. Nutritional counseling, individual nutritional measurement, and individual prediction of energy requirements might be necessary to prevent weight loss in this patient group.

## Supporting information

S1 Table Univariable logistic regression analysis for weight loss from hospital discharge to 6 months postoperatively. CI, confidence interval; OR, odds ratio; NE, not evaluable; BMI, body mass index; TG, total gastrectomy; DG, distal gastrectomy; PG, proximal gastrectomy; PPG, pylorus-preserving gastrectomy.
(DOCX)

S2 Table Multivariable logistic regression analysis for weight loss from hospital discharge to 6 months postoperatively. CI, confidence interval; OR, odds ratio; BMI, body mass index; TG, total gastrectomy; DG, distal gastrectomy; PG, proximal gastrectomy; PPG, pylorus-preserving gastrectomy.
(DOCX)

S3 Table Subgroup analysis for the effectiveness of nutrition counseling. BMI: body mass index, TG: total gastrectomy, DG: distal gastrectomy, PG: proximal gastrectomy, PPG: pylorus-preserving gastrectomy, CI: confidence interval. NE: not evaluable.
(DOCX)
