# Supplementary material for: Effectiveness of weight-loss prevention with continual nutrition counseling in postoperative outpatients with stage IA and IB gastric cancer
Source: PLoS One. 2023 Oct 19;18(10):e0292920. doi: 10.1371/journal.pone.0292920 (PMC10586603; doi:10.1371/journal.pone.0292920)
Supplement: S1 Table — CI, confidence interval; OR, odds ratio; NE, not evaluable; BMI, body mass index; TG, total gastrectomy; DG, distal gastrectomy; PG, proximal gastrectomy; PPG, pylorus-preserving gastrectomy. (DOCX) [file pone.0292920.s001.docx]

**S1** **Table. Univariable logistic regression analysis for weight loss from hospital discharge to 6 months postoperatively.**

| **Variable** | **Category**  **(Unit)** | **Weight loss of <5% Group** | **Weight loss of ≥5% Group** | **Univariable logistic model** | | |  |
| --- | --- | --- | --- | --- | --- | --- | --- |
|  |  | **（n=37）** | **（n=48）** | **OR** | **95% CI** | **p-value** | |
| Nutritional counseling group | One-time | 22 | 36 | 1 | － | － | |
|  | Four-times | 15 | 12 | 0.49 | 0.19-1.23 | 0.130 | |
| Age | (Age) | 64.9±11.1 | 69.3±10.6 | 1.04 | 0.10-1.08 | 0.076 | |
| Sex | Male | 24 | 27 | 1 | － | － | |
|  | Female | 13 | 21 | 0.70 | 0.29-1.69 | 0.422 | |
| Weight at discharge | (kg) | 55.3±11.7 | 56.7±9.4 | 1.01 | 0.97-1.06 | 0.552 | |
| BMI at discharge | (kg/m2) | 20.9±3.5 | 22.3±2.5 | 1.17 | 1.00-1.36 | **0.046** | |
| Weight loss rate from surgery to hospital discharge | (%) | -5.7±3.0 | -4.2±2.8 | 1.21 | 1.03-1.42 | **0.024** | |
| Gastrectomy extent | TG and PG | 6 | 17 | 1 | － | － | |
|  | DG and PPG | 31 | 31 | 0.35 | 0.12-1.01 | **0.053** | |
| Surgical approach | Laparotomy | 6 | 4 | 1 | － | － | |
|  | Laparoscopic/robotic | 31 | 44 | 2.13 | 0.55-8.18 | 0.271 | |
| Stage | I A | 29 | 37 | 1 | － | － | |
|  | I B | 8 | 11 | 1.08 | 0.38-3.03 | 0.887 | |
| Pretreatment | None | 29 | 42 | 1 | － | － | |
|  | Yes | 7 | 4 | 0.40 | 0.11-1.47 | 0.166 | |
| Diabetes | No | 30 | 43 | 1 | － | － | |
|  | Yes | 7 | 5 | 0.50 | 0.14-1.72 | 0.270 | |
| Chronic kidney disease | No | 36 | 48 | NE | － | － | |
|  | Yes | 1 | 0 | NE | NE | 0.991 | |
| Duration of hospitalization | (days) | 14.2±5.7 | 12.3±3.6 | 0.90 | 0.81-1.01 | 0.074 | |
| Albumin at discharge | (g/dl) | 3.4±0.4 | 3.3±0.4 | 0.43 | 0.11-1.67 | 0.222 | |
| C-reactive protein at discharge | (mg/dL) | 2.6±2.6 | 3.9±3.0 | 1.18 | 0.99-1.41 | 0.059 | |
| Total protein at discharge | (g/dl) | 7.0±0.4 | 7.0±0.6 | 0.92 | 0.41-2.08 | 0.840 | |
| Hemoglobin at discharge | (g/dl) | 12.2±1.5 | 12.2±1.5 | 0.98 | 0.73-1.30 | 0.868 | |
| Total lymphocytes at discharge | (/μl) | 1203.2±393.6 | 1211.7±330.4 | 1.00 | 1.00-1.00 | 0.913 | |

CI, confidence interval; OR, odds ratio; NE, not evaluable; BMI, body mass index; TG, total gastrectomy; DG, distal gastrectomy; PG, proximal gastrectomy; PPG, pylorus-preserving gastrectomy.
