# Supplementary material for: Effectiveness of weight-loss prevention with continual nutrition counseling in postoperative outpatients with stage IA and IB gastric cancer
Source: PLoS One. 2023 Oct 19;18(10):e0292920. doi: 10.1371/journal.pone.0292920 (PMC10586603; doi:10.1371/journal.pone.0292920)
Supplement: S2 Table — CI, confidence interval; OR, odds ratio; BMI, body mass index; TG, total gastrectomy; DG, distal gastrectomy; PG, proximal gastrectomy; PPG, pylorus-preserving gastrectomy. (DOCX) [file pone.0292920.s002.docx]

**S2** **Table. Multivariable logistic regression analysis for weight loss from hospital discharge to 6 months postoperatively.**

| **Variable** | **Category**  **(Unit)** | **Multivariable logistic analysis** | | |
| --- | --- | --- | --- | --- |
|  |  | **aOR** | **95% CI** | **p-value** |
| Nutritional counseling group | One-time | 1 |  |  |
|  | Four-times | 0.62 | 0.16-2.40 | 0.489 |
| Duration of hospitalization | (days) | 0.87 | 0.74-1.01 | 0.071 |
| Albumin at discharge | (%) | 0.44 | 0.08-2.30 | 0.329 |
| BMI at discharge | (kg/m2) | 1.21 | 0.96-1.53 | 0.099 |
| Percent reduction from preoperative to discharge | (%) | 0.67 | 0.51-0.89 | 0.006 |
| Gastrectomy extent | TG and PG | 1 | － | － |
|  | DG and PPG | 0.27 | 0.06-1.22 | 0.089 |

CI, confidence interval, aOR, adjusted odds ratio, BMI, body mass index, TG, total gastrectomy; DG, distal gastrectomy; PG, proximal gastrectomy; PPG, pylorus-preserving gastrectomy.
