# Supplementary material for: Effectiveness of weight-loss prevention with continual nutrition counseling in postoperative outpatients with stage IA and IB gastric cancer
Source: PLoS One. 2023 Oct 19;18(10):e0292920. doi: 10.1371/journal.pone.0292920 (PMC10586603; doi:10.1371/journal.pone.0292920)
Supplement: S3 Table — BMI: body mass index, TG: total gastrectomy, DG: distal gastrectomy, PG: proximal gastrectomy, PPG: pylorus-preserving gastrectomy, CI: confidence interval. NE: not evaluable. (DOCX) [file pone.0292920.s003.docx]

**S3** **Table.** **Subgroup analysis for the effectiveness of nutrition counseling.**

| **Variable** | **Category** | **Adjusted**  **RR** | **95% CI** | **p-value** | **p-value of**  **interaction**  **term** |
| --- | --- | --- | --- | --- | --- |
| Age | <65 years | NE | NE | NE | NE |
|  | ≥ 65 years | 0.34 | 0.05-2.23 | 0.263 |  |
| Sex | Male | 0.55 | 0.08-3.87 | 0.548 | 0.959 |
|  | Female | 1.90 | 0.08-45.20 | 0.691 |  |
| BMI at discharge | <23 kg/m^2^ | 3.32 | 0.23-47.02 | 0.375 | **0.014** |
|  | ≥ 23 kg/m^2^ | 0.09 | 0.01-1.26 | 0.083 |  |
| Gastrectomy extent | TG and PG | 1.83 | 0.10-32.49 | 0.679 | 0.953 |
|  | DG and PPG | 1.36 | 0.16-11.15 | 0.777 |  |
| Surgical approach | Laparotomy | NE | NE | NE | NE |
|  | Laparoscopic/robotic | 0.55 | 0.12-2.56 | 0.443 |  |
| Stage | I A | 0.51 | 0.12-2.19 | 0.366 | NE |
|  | I B | NE | NE | NE |  |
| Diabetes | No | 1.26 | 0.23-6.78 | 0.789 | NE |
|  | Yes | NE | NE | NE |  |
| Duration of hospitalization | <12 days | 1.35 | 0.06-33.13 | 0.852 | 0.622 |
|  | ≥12 days | 1.31 | 0.17-10.11 | 0.797 |  |

CI, confidence interval, RR, risk ratio, BMI: body mass index, TG: total gastrectomy, DG: distal gastrectomy, PG: proximal gastrectomy, PPG: pylorus-preserving gastrectomy, NE: not evaluable.
